# Innovations in Clinical Maxillofacial Tissue Engineering and Reconstruction: Cellular Bone Matrix Allografts, Autografts, and Growth Factors

**DOI:** 10.3390/cmtr19020018

**Published:** 2026-03-24

**Authors:** Jeffrey S. Marschall

**Affiliations:** Department of Oral and Maxillofacial Surgery, University of Iowa Hospital and Clinics, Iowa City, IA 52242, USA; jeffrey-marschall@uiowa.edu

**Keywords:** tissue engineering, bone grafting, cellular bone matrix, growth factors

## Abstract

Reconstruction of craniomaxillofacial (CMF) bony defects requires individualized strategies based on defect characteristics and graft bed biology, with traditional approaches relying on autogenous non-vascularized bone grafts or vascularized free flaps that, while reliable, are associated with donor-site morbidity and operative complexity. Biologically driven reconstructive strategies, including tissue engineering, cellular bone matrix allografts (CBMs), and growth factor adjuncts, have emerged as alternatives or complements to autograft-based reconstruction. This review introduces and details these new innovations with emphasis on the current literature, thus empowering surgeons to enhance their clinical armamentarium.

## 1. Introduction

Regeneration and reconstruction of bone is a central focus for craniomaxillofacial (CMF) surgeons. The etiology, size, location, and tissues removed and/or missing guide the reconstructive approach. Reconstruction of the craniofacial complex can consist of non-vascularized bone grafts and vascularized tissue transfer. Recently, tissue engineering and allogenic grafts have risen as alternatives for bony reconstruction of the face.

Tissue engineering is the use of a scaffold, cells, and bioactive molecules, such as growth factors, to promote regeneration of a target tissue—specifically, in this case, bone. Two major benefits of using tissue engineering techniques and allogenic components are a possible decrease in operative time and eliminating the need for a second surgical site. Current methods of maxillofacial tissue engineering have been referred to as “in situ tissue engineering” [1,2,3]. In this scenario, the matrix is generally supplied using allogenic bone, which is osteoconductive; stem cells are provided by a bone marrow aspirate concentrate, which is osteogenic; and the bioactive molecule (growth factor) is rhBMP-2, which is osteoinductive [1,2,3]. Notably, as with the aforementioned method, a second minor surgical site is required, and multiple adjunctive materials are used. Ideally, a single reconstructive material would be osteoconductive, osteogenic, and osteoinductive. An allogenic material that provides all three components is the cellular bone matrix allograft (CBM, e.g., ViviGen™, LifeNet Health, Virginia Beach, VA, USA). CBMs have been demonstrated to have viable, lineage-committed bone-forming cells within a bony matrix; bone morphogenetic proteins (BMP)-2 and BMP-7; vascular endothelial growth factor (VEGF); and angiogenin [4].

There have been few studies demonstrating the use of CBMs for maxillofacial tissue engineering [5,6,7]. The purpose of this article is to demonstrate the effectiveness of CBM-based maxillofacial tissue engineering by reviewing the current literature and providing case examples.

## 2. Cellular Bone Matrix Allografts

Traditional mandibular reconstruction had relied heavily on autologous vascularized bone flaps such as fibula free flaps [8]. These flaps have been reconstructive surgeons’ go-to due to reliable revascularization for composite defects. However, donor-site morbidity, operative complexity, and limitations in certain patient populations have motivated the search for alternative biomaterials.

Tissue engineering approaches aim to provide a biologically active scaffold capable of supporting new bone formation while minimizing surgical morbidity. Three major strategies dominate current clinical efforts: 1. cellular bone matrix allografts, which deliver viable osteogenic cells within a preserved extracellular matrix scaffold; 2. non-vascularized bone-grafting techniques that leverage autologous or allogenic bone; and 3. adjunctive biological agents—most notably bone morphogenetic proteins (BMPs)—that stimulate osteoinduction and enhance graft incorporation. Together, these strategies represent a continuum of biologically driven mandibular reconstruction modalities that may augment or replace traditional mandibular reconstruction.

Cellular bone matrices (CBMs) represent a major evolution in allogeneic graft technology and have gained increasing attention as potential alternatives to autogenous bone grafts for maxillofacial reconstruction. Unlike traditional demineralized bone matrix (DBM) or mineralized allograft chips, CBMs are processed to preserve living-donor-derived mesenchymal cells (MSCs) or lineage-committed bone cells (Osteoblasts and Osteocytes) within the native extracellular matrix. These constructs deliver all three essential elements of bone regeneration—osteoconduction, osteoinduction, and osteogenesis—positioning them as a promising modality for craniomaxillofacial applications where volume stability and biologic integration are critical. Commercial CBMs such as Osteocel^TM^ (Globus Medical, Audubon, PA, USA), ViviGen^TM^ (Virginia Beach, VA, USA), and Trinity Elite^TM^ (Orthofix Medical, Lewisville, TX, USA) have been used extensively in spine and orthopedic surgery [9,10,11,12], and increasingly in craniofacial applications [5,6].

**Figure 1:**
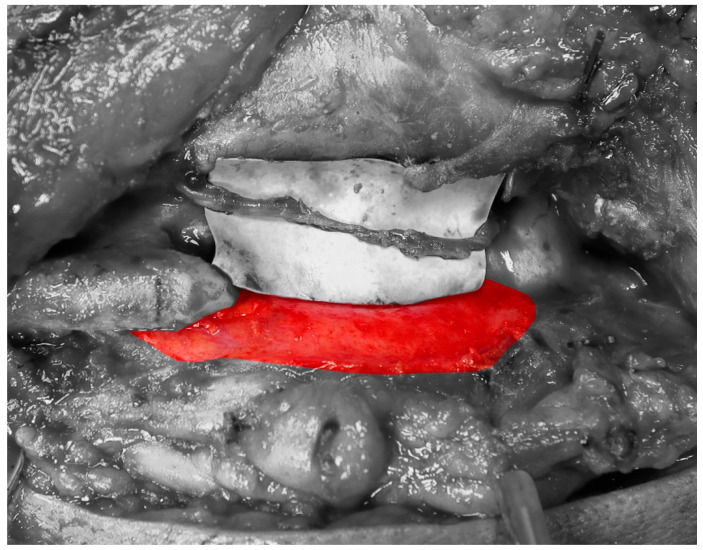
Highlighted area demonstrates healthy periosteum. Graft bed vasculature is essential for mandibular tissue engineering. Radiation and/or composite defects can jeopardize the quantity and quality of the graft bed.

The biologic basis for CBM or non-vascular bone graft use is simple: the soft tissue bed must be adequate in both quality and quantity (Figure 1) [7,13,14,15]. Large composite defects and sites that have had previous radiation treatment are contraindications to this reconstructive modality due to inadequate soft tissue and destruction of the microvasculature that will support the CBM or graft. Successful graft incorporation requires rapid revascularization and the recruitment of osteogenic cells capable of producing woven and lamellar bone. CBMs are designed to provide an immediate reservoir of viable cells that may enhance early osteogenesis and accelerate graft integration compared with acellular allografts [16].

Despite CBMs stemming from the same fundamental idea—that is, an allogenic source of osteogenic cells—CBMs can differ substantially across multiple practical and biologic dimensions. These differences influence handling characteristics and cell type; understanding these differences is essential when selecting a CBM for mandibular tissue engineering.

One difference is the composition of the bone matrix that carries the viable cells. Some CBMs are formulated primarily from cortical cancellous allograft chips (e.g., Vivigen^TM^, Virginia Beach, VA, USA). To enhance handling characteristics, some products contain demineralized fibers (Vivigen Formable^TM^, Virginia Beach, VA, USA; Trinity Elite^TM^ Orthofix Medical, Lewisville, TX, USA). These products are designed to be easily packed into irregular alveolar or cystic defects and to remain cohesive within titanium mesh constructs. Some manufacturers offer both a solid chip form and a formable variant achieved by adding a carrier that confers malleability without significantly altering osteoconductivity. It is this author’s preference to use CBMs that have a fiber component as the handling characteristics are excellent [5,6].

Manufacturing processes are another major point of divergence. CBMs are typically processed using proprietary aseptic techniques prior to cryopreservation. Manufacturers utilize controlled-rate freezing with cryoprotectants (for example, dimethyl sulfoxide, DMSO) or other proprietary formulations; controlled-rate freezing may help minimize cell damage from osmotic membrane imbalance and may reduce ice crystal formation within the cell [17]. For long-term storage prior to shipping to the hospital or point of care, CBMs are maintained in vapor-phase liquid nitrogen tanks, usually around −185 degrees Celsius. Shipping occurs as quickly as possible, usually next-day shipping, with dry ice and special packaging. CBMs are generally maintained in the tissue banks of the medical center at at least −70 degrees Celsius. To maintain cellular viability within the CBMs, rapid-thaw processes are usually implemented. The process is manufacture-specific; therefore, it is important to know the specifics of the product.

Perhaps the most profound differences among CBMs relate to the cellular populations in the product. Some products describe their cellular component as a mixture of viable mesenchymal stromal cells (MSCs), osteoprogenitors, and osteoblast-lineage cells (e.g., Osteocel^TM^ Globus Medical, Audubon, PA, USA); others emphasize lineage-committed bone cells rather than multipotent MSCs (ViviGen^TM^, Virginia Beach, VA, USA). The distinction matters: MSCs are multipotent and can contribute to osteogenesis (or adipogenesis), whereas lineage-committed bone cells may be more immediately osteogenic but potentially less proliferative [18,19]. Reported post-thaw cell viabilities and cell counts vary both between products and between lots of the same product. There is a range of viable cell yields, with some products reporting higher viable cell counts and in vitro proliferative potential than others [20,21]. MSC-containing products generally have higher cell counts compared to lineage-committed cellular products, but the viability of these cells is lower [20,21]. It is important to note, however, that viability assays do not directly equate to clinical efficacy; viable cell counts are only one determinant of whether a CBM will contribute meaningfully to bone formation in vivo.

CBM safety is a major concern for manufactures, clinicians, and patients. This is influenced by donor screening, aseptic processing, and terminal sterilization (when applied). CBMs generally avoid terminal sterilization to preserve cell viability and are therefore processed aseptically with validated sterility testing. The absence of terminal sterilization raises theoretical infection concerns; in practice, published surveillance and registry data report low rates of infectious transmission with modern tissue banking and donor-screening protocols, although vigilance is warranted [17,22]. Immunogenicity is typically low because mesenchymal-lineage cells express low levels of HLA class II and have immunomodulatory properties; nevertheless, product processing that depletes hematopoietic cells is often highlighted by manufacturers to minimize immune activation [17,22,23].

Preserving viability necessitates cold-chain logistics and on-site thaw protocols. Some CBMs require ultra-low-temperature storage (−70 degrees Celsius) and are delivered on dry ice with immediate thawing during the procedure. Cost per unit varies considerably among products and often exceeds that of conventional allograft, with prices depending on hospital contracting and formulation (formable vs. particulate). Cost-effectiveness analyses are limited; clinicians must weigh the potential reduction in donor-site morbidity and operative time against higher graft acquisition costs.

**Figure 2:**
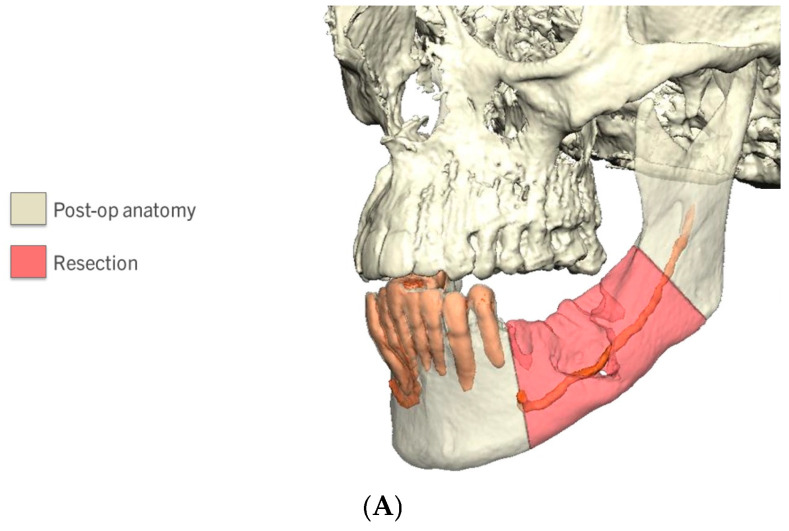

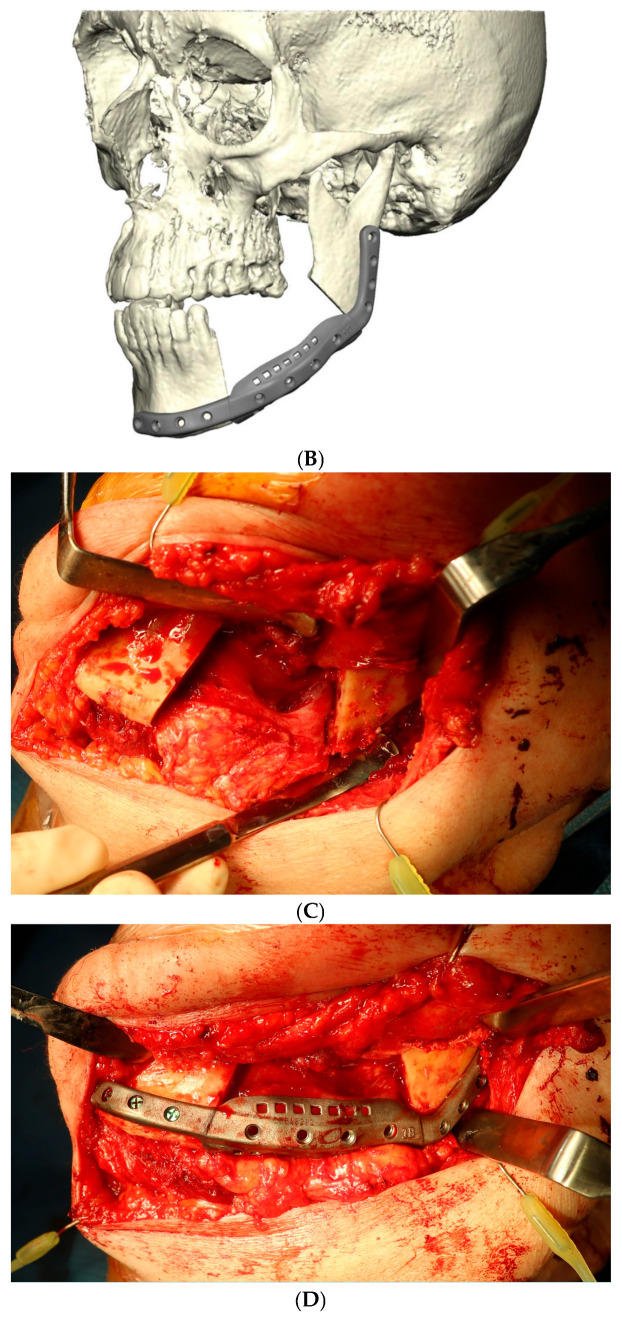

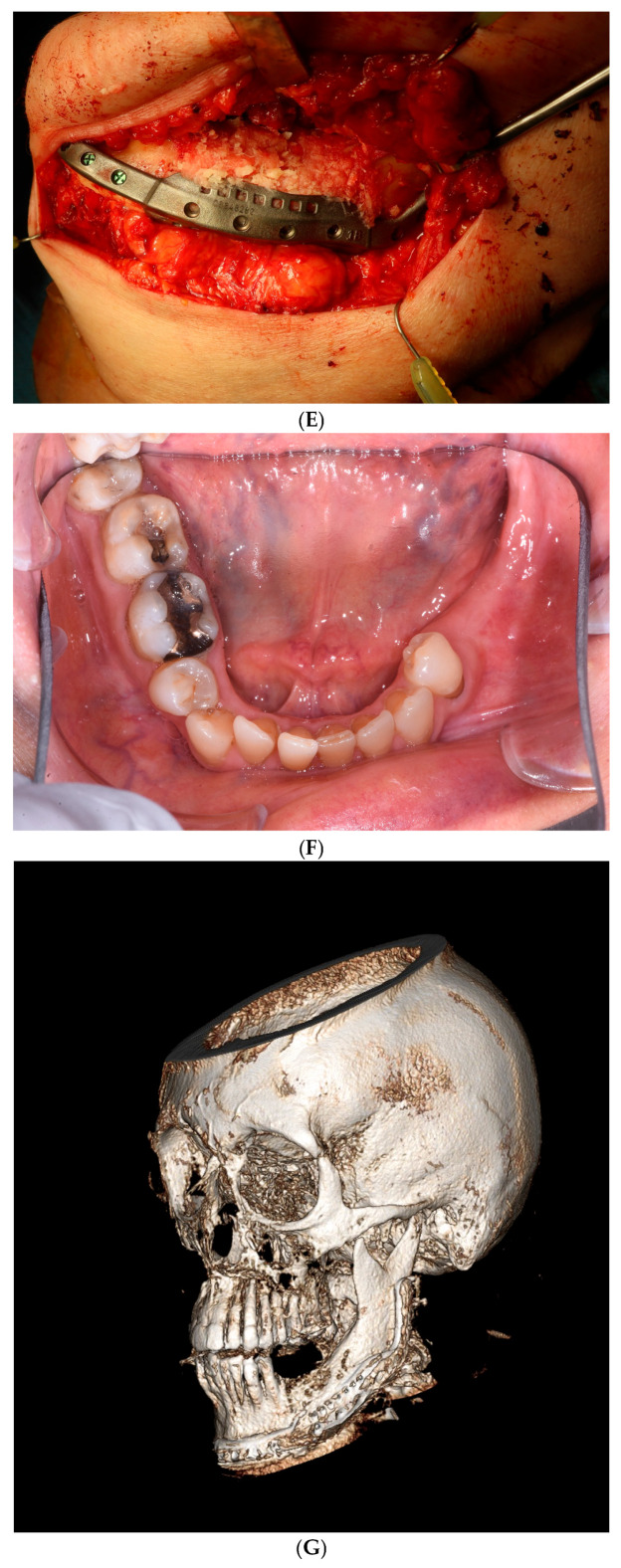

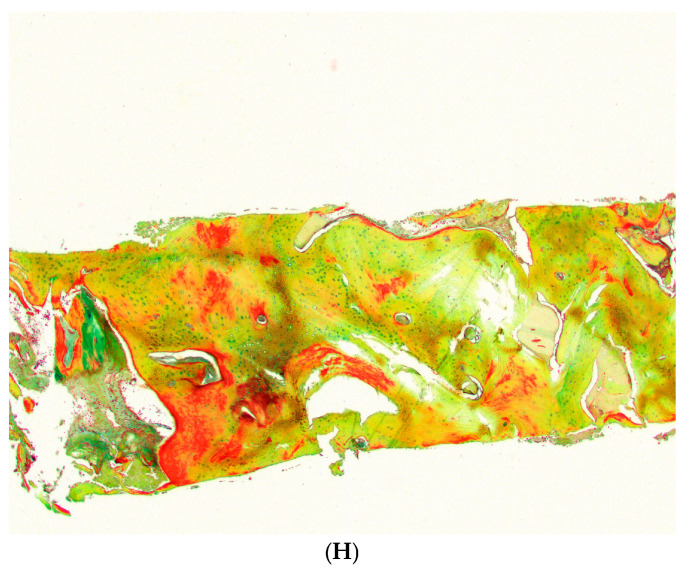
A 78-year-old female with left mandibular pathologic fracture secondary to osteomyelitis that was reconstructed with cellular bone matrix and rh-BMP-2. (**A**) Preoperative imaging from planning session demonstrating area of bone to be resected. (**B**) Resected bone with patient-specific locking reconstruction plate with mesh. (**C**) Clinical photograph of resected mandible and healthy soft tissue bed. (**D**) Patient-specific mandibular reconstruction plate in place. (**E**) Tissue engineering construct (cellular bone matrix and rh-BMP-2) placed into defect site. (**F**) Intra-oral photograph taken 11 months after resection. (**G**) Cone-beam computed tomography scan 3D reconstruction 11 months after resection demonstrating excellent consolidation of bone. (**H**) Bone core taken during endosseous implant placement in a separate patient in a site reconstructed with cellular bone matrix; Orange/red = osteoid; yellow = calcified bone.

Selection of a CBM for mandibular tissue engineering should be individualized (Figure 2). Considerations include the defect biology and size (well-vascularized defects may be amenable to CBMs; large composite and/or radiated defects typically require vascularized transfer) and the available clinical evidence for the anatomic site. In practice, CBMs are frequently chosen when the goal is to avoid autograft harvest for lateral/linear continuity defects, whereas surgeons confronting larger mandibular continuity defects may rely on hybrid approaches combining CBMs with autograft [5,7].

Overall, the literature for CBM-based mandibular reconstruction and tissue engineering is limited but growing. The largest series by Marschall et al. [5] included 38 subjects. In total, 50% of the subjects were reconstructed with CBM plus an autograft. Overall success of the reconstructions was 73.7%. Interestingly, subjects with a reported penicillin allergy or a history of diabetes had significantly lower success [5]. This investigation demonstrated the viability of CBM/autograft combination and CBM-based mandibular reconstruction for the first time. There have been several other case reports demonstrating success in geriatric and pediatric patients as well [24,25].

**Figure 3:**
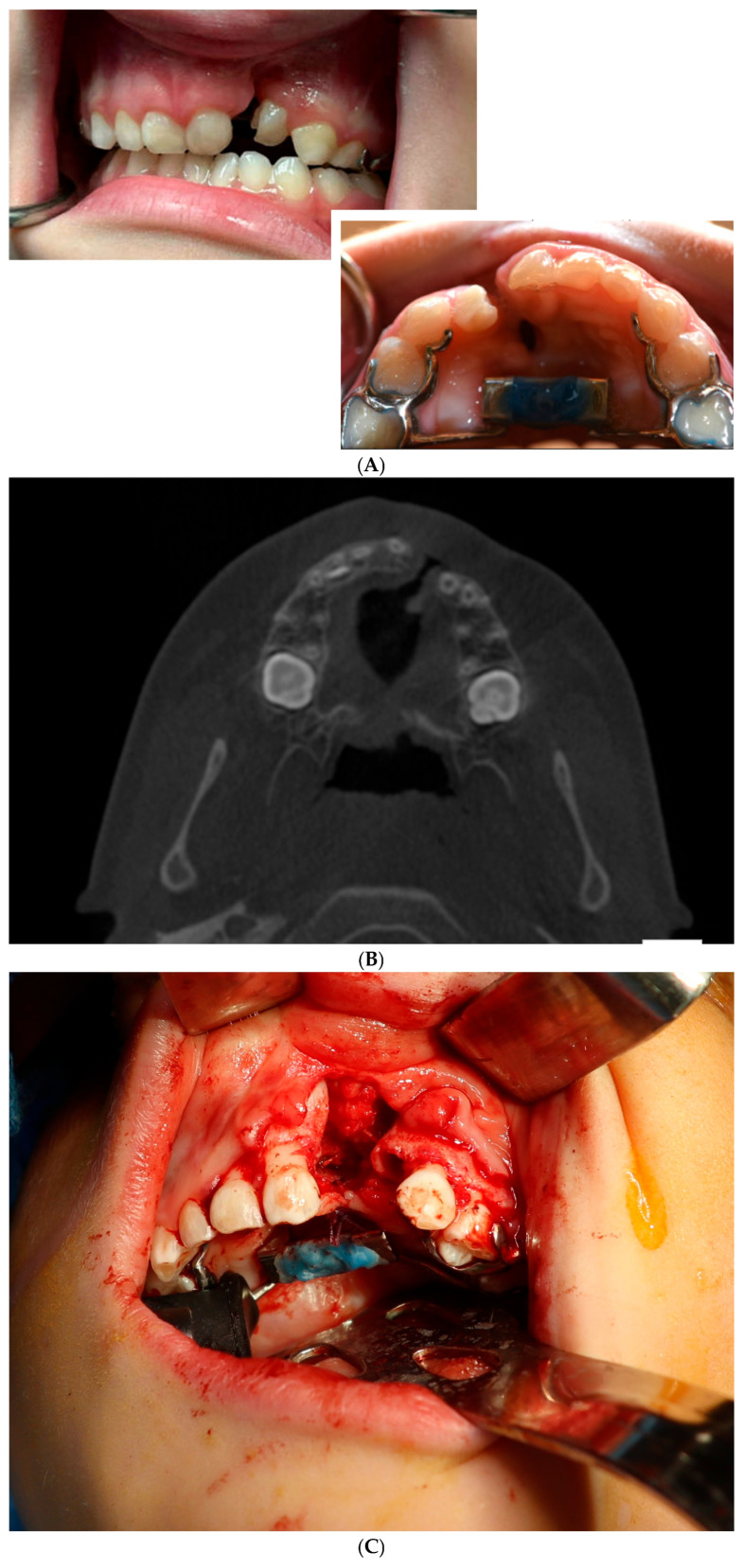

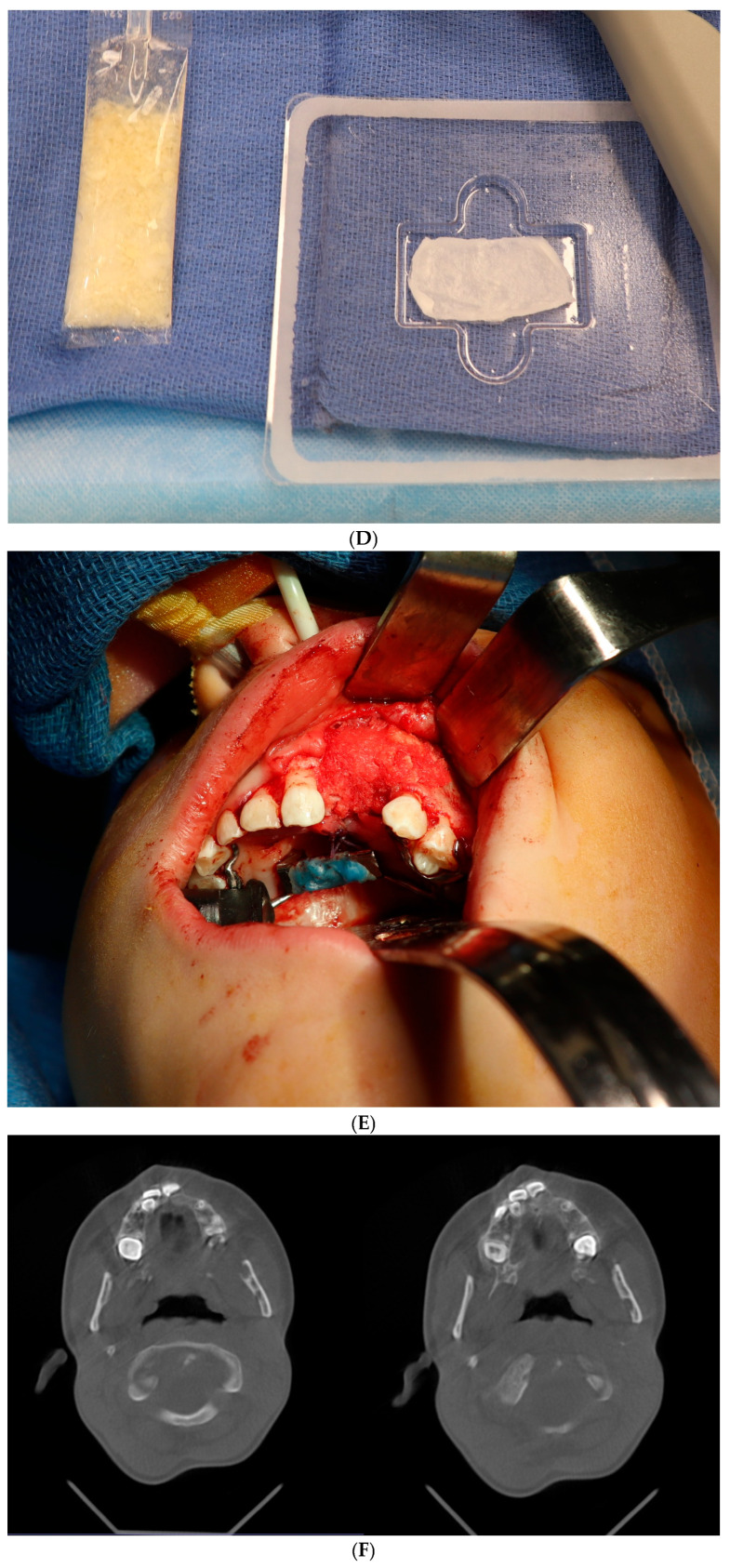

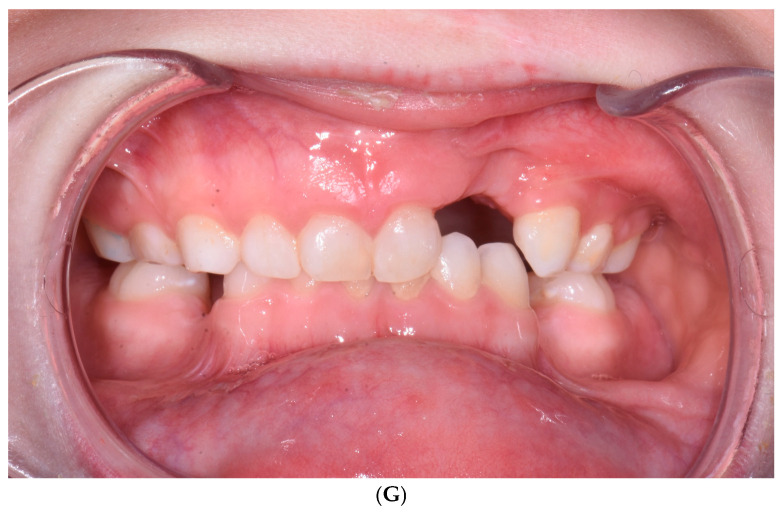
A 6-year-old male with left complete cleft lip and palate that was reconstructed with cellular bone matrix and rh-BMP-2. (**A**) Pre-operative intra-oral photographs. (**B**) Axial cone-beam computed tomography scan demonstrating cleft. (**C**) Intra-operative photograph demonstrating fistula repair and cleft defect. (**D**) Photograph of tissue engineering materials: left—cellular bone matrix, right—rh-BMP-2 sponge. (**E**) Tissue engineering construct placed into cleft maxilla. (**F**) Axial CT scan taken 6 months after surgery demonstrating excellent continuity of the maxilla. (**G**) Intra-oral photograph taken 6 months after surgery demonstrating excellent healing.

CBM-based reconstruction has also found a niche in maxillary bone defect repair (Figure 3). A recent report demonstrated 87.5% success in 48 subjects. Most of the defects reported in this study were sites reconstructed after extirpation of odontogenic cysts, representing simple bone defects. However, CBMs have been used in cleft orthognathic surgery, and in the management of bilateral oro-sino orbital fistula in bilateral Tessier IV clefts [26,27]. Another intriguing application of CBMs is in cleft maxilla reconstruction [28]. Indeed, this is one of the areas where the author is using this reconstructive modality the most.

CBMs will likely play a larger role in craniomaxillofacial reconstruction. Further investigations are needed to determine the limits of their use. Regardless, the fundamental biologic principles pertaining to their success are similar to those of autograft, which will be discussed next.

## 3. Autografts

**Figure 4:**
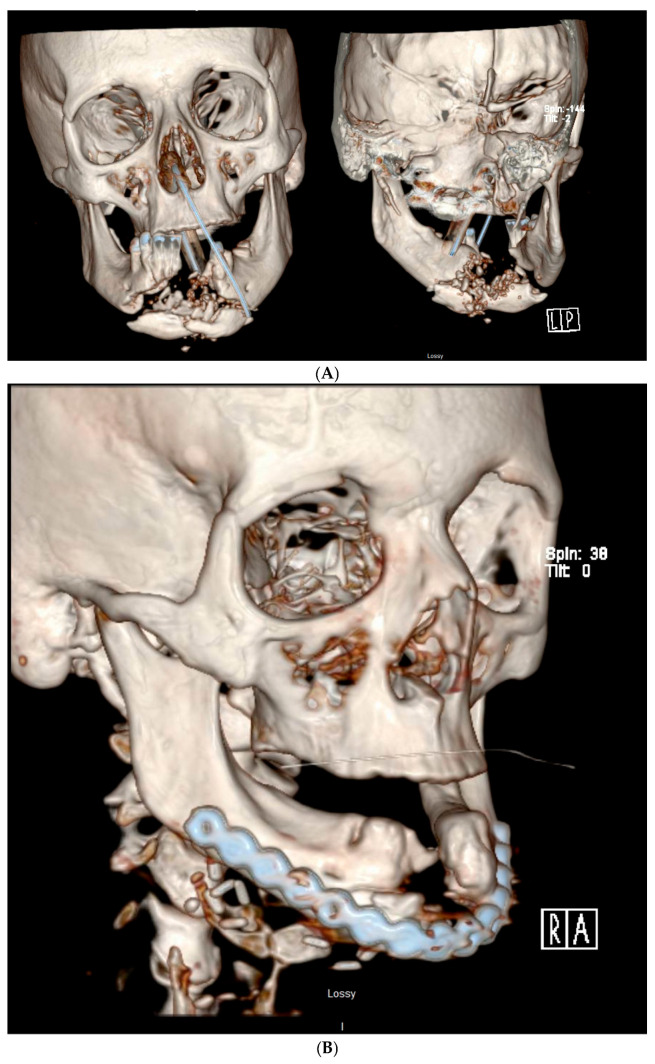

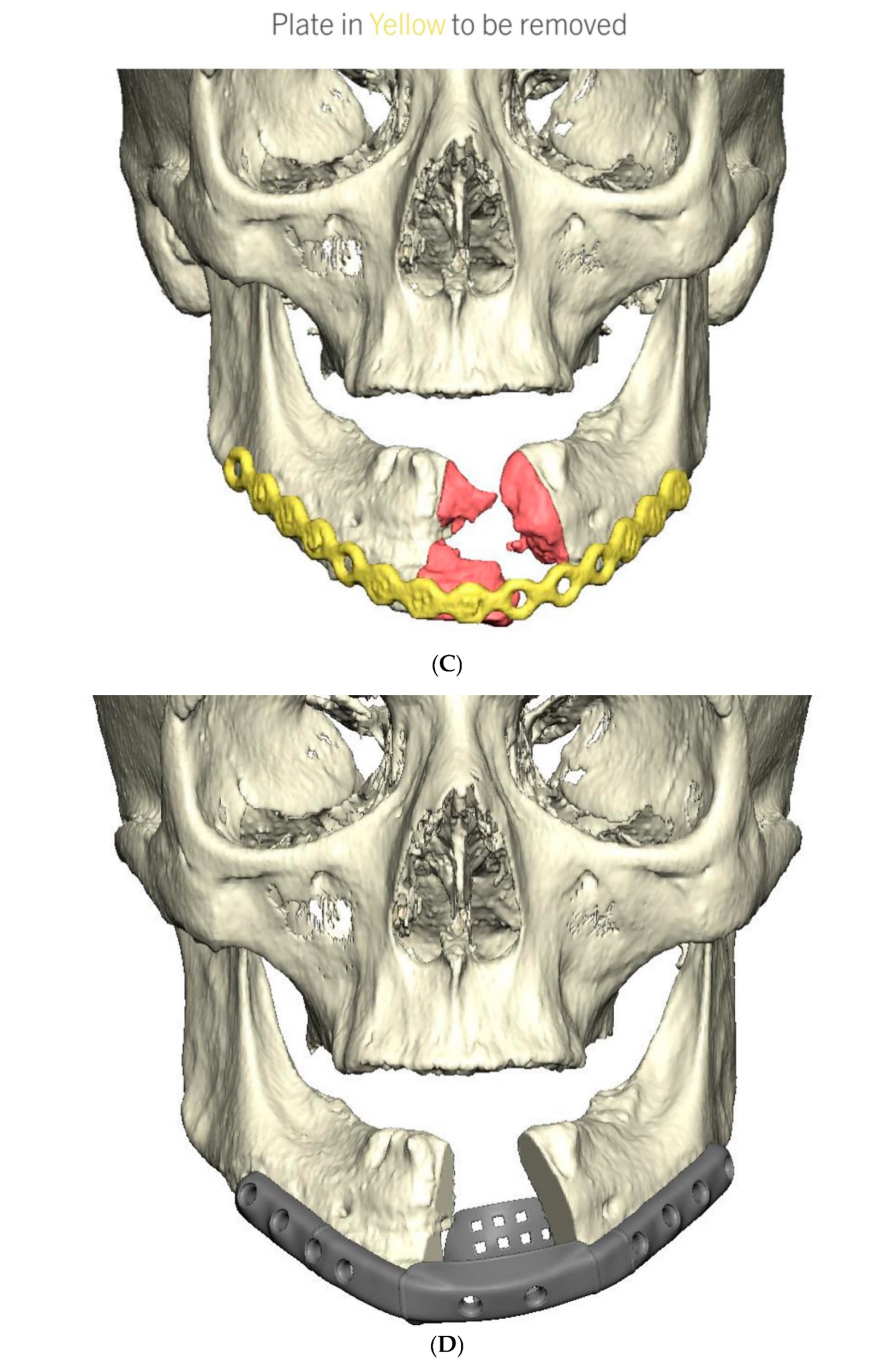

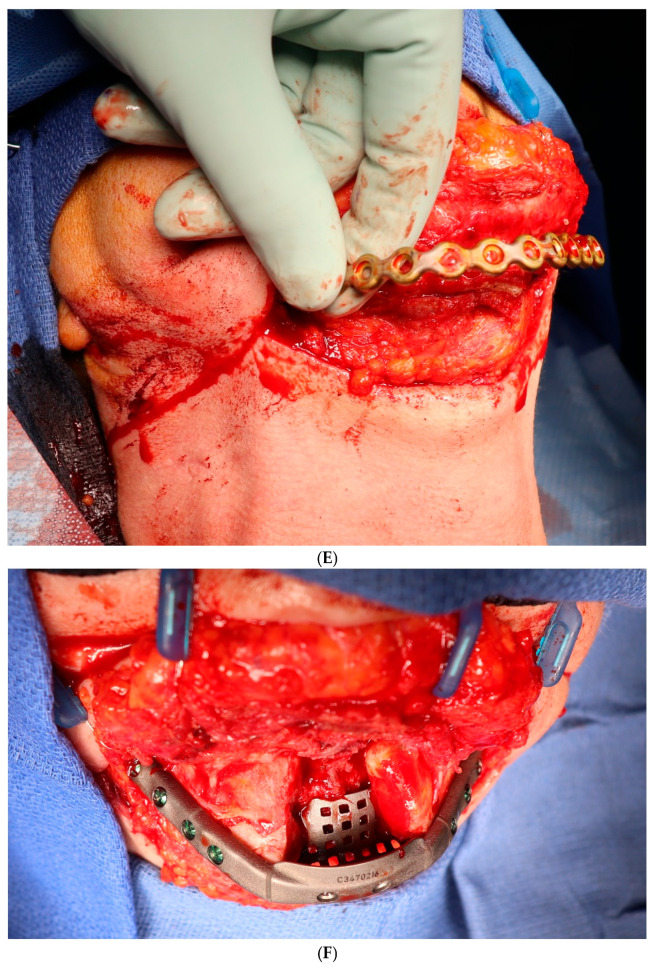

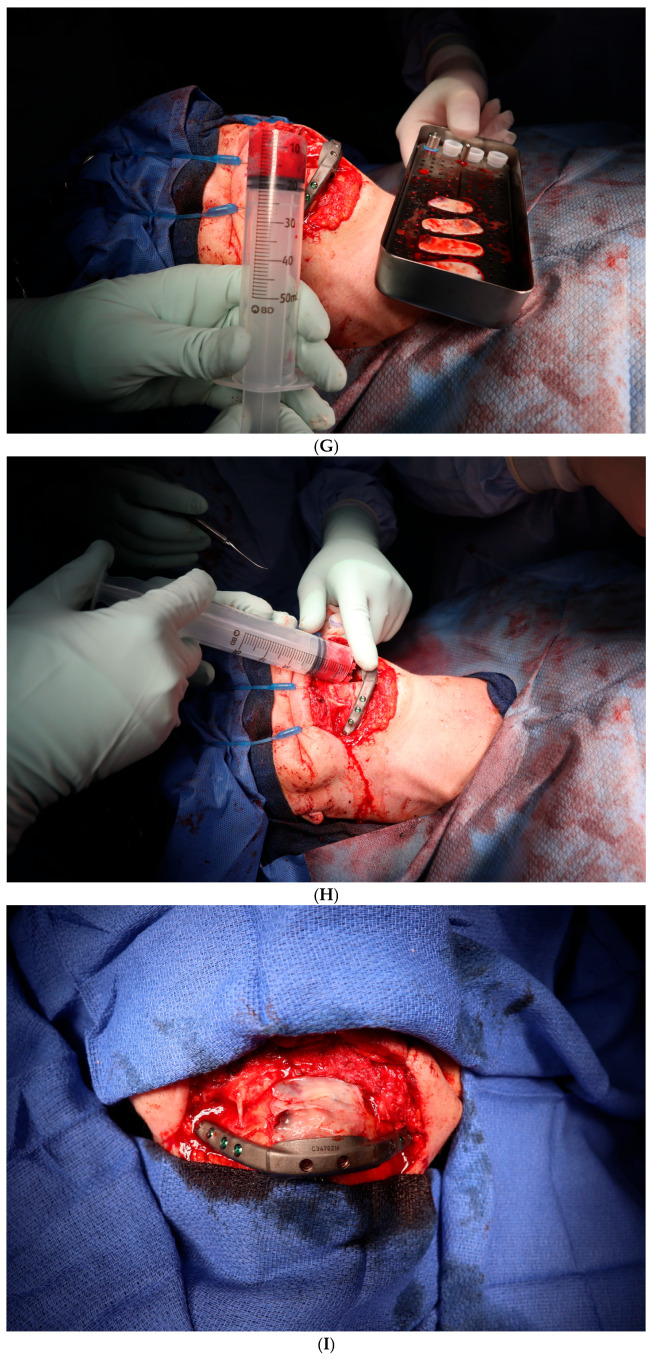

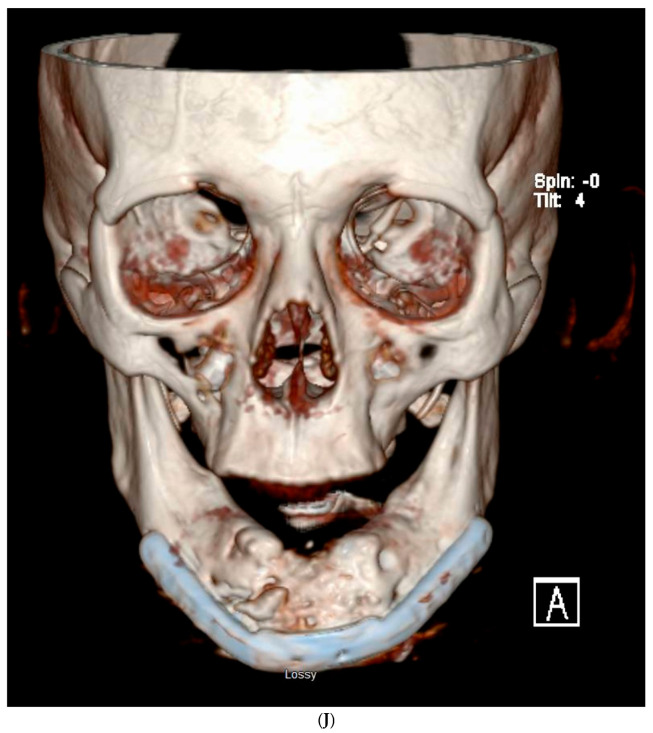
A 44-year-old female that was shot in the face that had original reconstruction plate removed secondary to intra-oral exposure and infection with reconstruction with autogenous bone graft and platelet-rich fibrin. (**A**) Initial CT scan demonstrating mandibular fracture. (**B**) Post-operative CT scan completed by another surgeon demonstrating continuity defect. (**C**) Planned removal of infected plate and bone. (**D**) Patient-specific reconstruction plate and titanium crib. (**E**) Intra-operative photograph demonstrating infected-plate removal. (**F**) Patient-specific reconstruction plate in place. (**G**) Autogenous bone graft in syringe and platelet-rich fibrin membranes. (**H**) Bone graft being placed into defect. (**I**) Bone graft in place with platelet-rich fibrin membranes over graft. (**J**) Post-operative CT scan demonstrating reconstruction of mandibular continuity defect.

Non-vascularized bone grafts are still an excellent source of cells and bone for mandibular reconstruction. While CBMs are challenging the status quo, autogenous bone remains the gold standard (Figure 4). Non-vascular free bone grafts have traditionally been used for reconstruction of mandibular defects less than 6 cm in length in healthy soft tissue beds that have not been subjected to radiation [15,29]. Recently, the long-standing “6 cm rule” is being challenged [2,7,30,31,32]. Autogenous non-vascular bone grafts can be harvested as cancellous bone and marrow and cortical and corticocancellous bone. Common locations include the iliac crest and proximal tibia. The tibia bone graft is an excellent source for cancellous bone and is the author’s go-to for adult non-vascular bone harvest. This procedure is relatively easy to perform with a low complication rate [33]. Twenty-five cubic centimeters of cancellous bone can be harvested from a single site [33]. Interestingly, an investigation by Engelstad and Morse demonstrated that the proximal tibia yielded a significantly greater mean volume of compressed cancellous bone when compared to the anterior iliac crest [34]. Cancellous bone from the proximal tibia has been demonstrated to be a predictable graft for a variety of mandibular defects, both large and small [5,7,30,35].

As mentioned above, surgeons are challenging the “6 cm rule”. Deeper understanding of bone graft biology and new adjuncts are extending the predictability of non-vascularized bone regeneration and reconstruction. Nandra et al. [31] reported that 13 of 14 bone grafts were successful in defects greater than 6 cm. Schlieve et al. [32] reported that of 11 grafts larger than 6.38 cm, 9 were successful and 2 were failures. Marechek et al. [14] added further evidence that non-vascular bone grafts are effective for defects greater than 6 cm. Furthermore, another author has demonstrated predictable reconstruction with autograft alone, CBMs, and growth factors for large segmental defects [5,7,30]. Melville et al. has also demonstrated large mandibular reconstruction via “in situ” tissue engineering methods, which use a combination of rhBMP-2, bone marrow aspirate concentrate, and off-the-shelf allograft [1,2,3,36]. A common thread from all these studies is that none of the patients had undergone radiotherapy, demonstrating the importance of the graft bed biology and healthy microvasculature.

## 4. Growth Factors

Bone regeneration is a dynamic biological process that occurs in three stages: an initial inflammatory response, a subsequent reparative phase, and a final period of remodeling [35,37]. Progression through these stages depends on tightly regulated interactions between signaling molecules and the cellular populations they recruit. Because growth factors play a central role in coordinating inflammation, angiogenesis, and osteogenesis, they have been widely investigated as therapeutic adjuncts to enhance bone healing. Beyond their role in skeletal repair, growth factors govern fundamental cellular behaviors—including proliferation, migration, and lineage commitment—making controlled delivery strategies particularly relevant in regenerative medicine. In oral and maxillofacial surgery, clinically utilized growth factors are generally divided into autologous, blood-derived biologic modifiers and exogenously produced recombinant proteins.

Among autologous biologics, platelet-rich plasma (PRP) represents one of the earliest growth-factor-based therapies to gain broad clinical acceptance [38]. PRP is readily obtained via routine venipuncture and contains multiple signaling molecules with known roles in tissue repair, including platelet-derived growth factor, transforming growth factor-β, fibroblast growth factor, insulin-like growth factors 1 and 2, and vascular endothelial growth factor [39,40]. Despite these advantages, PRP is limited by considerable interpatient variability and a rapid, short-lived release of growth factors. In physiologic wound healing, growth factors are embedded within the extracellular matrix and exert their effects over extended periods, often lasting days to weeks [41]. Platelet-rich fibrin (PRF) was subsequently introduced to better approximate this natural release profile (Figure 4) [42]. Contemporary tissue engineering principles emphasize the importance of a three-dimensional scaffold capable of supporting cellular infiltration and differentiation, ideally with incorporated biochemical cues. In PRF, a fibrin-based matrix functions as both a scaffold and a reservoir for platelets and leukocytes, creating a biologically active construct rich in growth factors. The entrapment of these signaling molecules within the fibrin network results in more sustained and physiologically relevant release kinetics [40]. These features have contributed to the expanding clinical use of PRF in procedures such as reconstruction following extensive mandibular resection [5,13,30].

Bone morphogenetic proteins (BMPs) were first described in the mid-twentieth century and are now recognized as members of the transforming growth factor-β superfamily. This group consists of approximately 20 related proteins with demonstrated roles in angiogenesis, inflammation, programmed cell death, and the formation of bone and cartilage [43]. BMP-2, in particular, has emerged as one of the most potent osteoinductive agents identified to date [44]. Its biological activity is mediated through binding to specific receptors on undifferentiated mesenchymal cells, triggering intracellular signaling via SMAD transcription factors [43]. Activation of this pathway promotes osteoblastic differentiation, directed cell migration, new bone formation, and synthesis of extracellular matrix [43]. The ability of BMP-2 to induce bone formation is especially valuable when used in conjunction with allograft or xenograft materials, which lack inherent osteoinductive properties; in this context, BMP-2 may enhance host cell infiltration and render these substitutes functionally closer to autologous bone [2,3,35,45,46,47,48]. Recombinant human BMP-2 (rhBMP-2; Infuse, Medtronic) is currently available for clinical use. Multiple studies have shown that rhBMP-2, delivered on an absorbable collagen sponge and combined with allo- and/or xenografts, achieves outcomes comparable to, and in certain scenarios superior to, autologous grafting in alveolar cleft repair [49] and reconstruction of critically sized defects of the maxilla and mandible [44,45]. In maxillofacial surgery, the most commonly described complications include pronounced post-operative edema, erythema, and dysphagia [49]; however, these effects are typically transient, rarely compromise overall graft success, and are likely due to supraphysiologic dosing. The author recommends not exceeding a medium-size BMP sponge. Indeed, an extra-extra-small or extra-small BMP sponge is adequate for most maxillofacial applications.

## 5. Conclusions

Reconstruction of maxillofacial bony defects requires careful consideration of defect characteristics, soft-tissue-bed biology, and the biologic properties of the reconstructive material. Autogenous bone grafting, cellular bone matrix allografts, and growth-factor-based adjuncts each represent distinct but overlapping strategies grounded in the fundamental principles of osteoconduction, osteoinduction, and osteogenesis. Autogenous non-vascularized bone grafts remain a reliable and well-established option for appropriately selected defects in well-vascularized tissue beds, and recent evidence suggests their utility may extend beyond traditionally accepted size limitations when host biology is favorable.

Cellular bone matrix allografts represent an evolution in allogeneic graft technology, offering the potential to deliver viable osteogenic cells within an extracellular matrix while avoiding donor-site morbidity. Although CBMs differ in matrix composition, cellular content, processing, and handling characteristics, early clinical experience in mandibular and maxillary reconstruction demonstrates that they may be effective in selected non-radiated defects, either alone or in combination with autograft. Growth factors, including platelet-derived products and recombinant BMPs, further augment reconstructive strategies by modulating the biologic environment and enhancing osteogenesis, particularly when used within appropriate dosing parameters.

**Figure 5:**
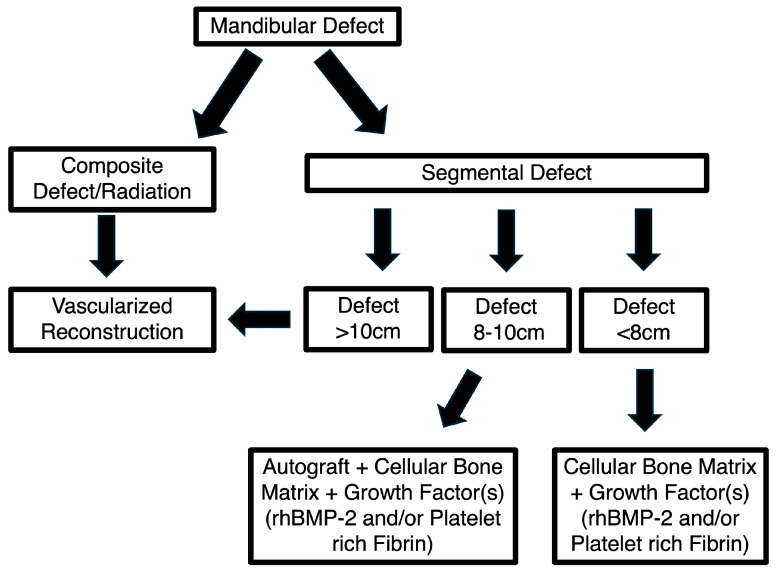
The author’s treatment algorithm for mandibular reconstruction.

The author’s preferred mandibular reconstructive strategy is summarized in Figure 5. In defects with compromised graft bed biology—such as those associated with prior radiation therapy and/or composite tissue loss—vascularized free tissue transfer is favored. For defects measuring <8 cm, a tissue engineering approach using CBM combined with a growth factor source (rhBMP-2 and/or PRF) is employed. For defects measuring 8–10 cm, autologous bone grafting from the proximal tibia or iliac crest is incorporated into the construct. For defects >10 cm, free tissue transfer remains the most reliable and predictable reconstructive modality [5,7,30].

Collectively, these biologically driven approaches expand the reconstructive armamentarium available to craniomaxillofacial surgeons and underscore the importance of defect-specific planning and soft-tissue-bed quality. While the existing clinical literature remains limited, current evidence supports the selective use of CBMs, autografts, and growth factor adjuncts as complementary tools. Continued clinical investigation and longer-term outcome data will further define their respective roles and limitations in craniomaxillofacial reconstruction.

## Data Availability

No new data were created or analyzed in this study.
